# Configuration Path Study of Influencing Factors on Health Information-Sharing Behavior among Users of Online Health Communities: Based on SEM and fsQCA Methods

**DOI:** 10.3390/healthcare11121789

**Published:** 2023-06-17

**Authors:** Minhao Xiang, Tianning Guan, Mengqi Lin, Yujie Xie, Xingyu Luo, Minghua Han, Kun Lv

**Affiliations:** Business School, Ningbo University, Ningbo 315211, China; 206002801@nbu.edu.cn (M.X.); 206001990@nbu.edu.cn (T.G.); 206003823@nbu.edu.cn (M.L.); 216001730@nbu.edu.cn (Y.X.); 206003727@nbu.edu.cn (X.L.); hanminghua@nbu.edu.cn (M.H.)

**Keywords:** online health community, health information, information sharing, influencing factors, configuration path

## Abstract

This study examines the determinants that drive the behavior of sharing health information within online health communities. Leveraging the Theory of Planned Behavior, the Technology Acceptance Model, and the “Knowledge-Attitude-Practice” theory, a comprehensive model elucidating the key elements that sway the health information-sharing behavior among users of online health communities is designed. This model is validated through Structural Equation Modeling (SEM) and Fuzzy Set Qualitative Comparative Analysis (fsQCA). Findings derived from the SEM suggest that perceived ease of use, perceived usefulness, perceived trust, and perceived behavioral control exert a significant positive impact on attitudes towards health information sharing, the intention to share health information, and the actual health information-sharing behavior. The fsQCA unfolds two unique configuration path models that lead to the emergence of health information-sharing behavior: one predicated on perceived trust and sharing intention, and the other on perceived usefulness, behavioral control, and sharing attitude. This research provides invaluable insights, fostering a deeper comprehension of the dynamics involved in health information sharing within online communities, thereby directing the design of more effective health platforms to augment user engagement and enable informed health decisions.

## 1. Introduction

Online health communities are web-based platforms that offer information services such as online search, access, sharing, and communication of health information for health enthusiasts and other community users [1]. In China, the major online health communities include specialized social health platforms such as “Baidu Health”, “Chunyu Doctor”, and “Meet you” [2]. With the support of the “Healthy China” strategy and policies such as “Internet + Medical Health,” online health communities have rapidly developed and are able to provide personalized health information services to different population groups based on their characteristics [3]. This provides an important platform for the public to participate in interactive discussions and share health information.

The sustained development of online health communities requires active participation from users, with sharing health information being a crucial aspect [4]. Sharing health information in the communities enables users to access solutions to their own health problems and, at the same time, health information itself has significant positive externalities that can promote the spread of health information and knowledge [5,6]. Therefore, user-sharing behavior is becoming a new channel for disseminating health and medical information and facilitating online interactions between doctors and patients [7]. One of the most important objectives of building online health communities is to increase user activity and health information-sharing intention in order to enhance the communities’ reputation and reach more users [8]. Exploring the factors that influence users’ health information-sharing behavior can help the communities take targeted measures to promote user communication and maintain their sustained participation, which is of great importance for the health operation of online health communities [9].

In recent years, health information sharing in online communities has attracted widespread scholarly attention, both nationally and internationally. This interest has led to the investigation of real-world scenarios within these communities and consequently, to the identification of several factors that influence health information sharing. For example, studies conducted by Maloney-Krichmar D [10] and Martijn V.D et al. [11] on online health community platforms suggest that factors such as social participation, empathy, and enjoyment can stimulate health information sharing. They found that patients often search for health information based on their symptoms within these communities. In further research Yan Z [12] and Hargreaves S et al. [13] examined health information-sharing behavior from a social-psychological perspective, focusing on its specific manifestations and influencing factors. It became clear that online health communities serve as an open network for users to exchange information, share experiences, partake in Question and Answer (Q&A) consultations, and garner social support concerning health and medical issues [14]. For the general public and patients, especially those with chronic diseases, the powerful communication and interaction function of online health communities has an important positive impact on their health self-management and daily disease control [15,16]. At the same time, online health communities are also beneficial to alleviate the practical problem of limited and uneven distribution of medical and health resources [17]. Insights from Zhang et al. [18] suggest that the dynamics of online health information sharing are shaped by an interplay of various factors. They found that physicians’ motivations to share health information for free are primarily driven by both material and professional interests. However, professional interests seem to dominate among physicians with advanced skills. Extending this notion, Meng et al. [19] indicated that a physician’s general knowledge-sharing behavior could influence their specific knowledge-sharing activities, a relationship that is strengthened by online reputation and patient involvement. Liu et al. [20] underscored the significant value of online health interactions to third-party patients, emphasizing the importance of prevention information, emotional support, and uncertainty in physicians’ responses to the perceived usefulness of the information. Guo et al. [21] presented a nuanced relationship between physicians’ online information sharing and patient education, where increased sharing augments potential education but induces an inverted U-shaped effect on realized education. These relationships are moderated by the physicians’ online reputation and offline expertise. Additional studies, such as Lei Y et al. [22], who analyzed user-generated content and related user behavior of two popular online health communities in China, provide valuable insights for optimizing treatment design and medical services. Zhang X et al. [23] explored the factors preceding and resulting from the privacy disclosure behavior of online health community users, discovering that users’ privacy concerns impact their willingness to disclose personal health information to varying degrees. Gabarron E et al. [24] analyzed the behavioral data from Wikipedia users retrieving health information and noticed a pattern of initial increase and subsequent decrease in retrievals over the workweek.

Research on online health information-sharing behavior has produced several influential models and adopted diverse methodologies. Some studies utilized theoretical approaches such as evolutionary game theory, while others opted for empirical analysis on actual data and case studies. A critical gap in the extant literature is the predominant focus on the impact of individual factors on health information-sharing behavior, overlooking the combined effects of multiple factors. Additionally, the intricate interrelationships among these factors, their impact hierarchy, and specific influence pathways are largely unexplored.

Addressing these gaps, this study offers three significant contributions. First, it transcends the focus on single influencing factors by integrating multiple factors affecting health information-sharing behavior in online health communities. This holistic approach is guided by the theory of planned behavior, the technology acceptance model, and the “knowledge-Attitude-Practice” theory. The aim is to develop an inclusive model of the factors influencing users’ health information-sharing behavior in online health communities, thereby expanding theoretical insights into this phenomenon. Second, this research invigorates methodological discourse by presenting a balanced fusion of qualitative and quantitative research approaches for examining the factors driving users’ health information-sharing behavior. This composite methodology, combining statistical quantitative analysis with fuzzy set qualitative comparative analysis (fsQCA), counters the one-dimensionality of single-method research, furnishing a more nuanced and comprehensive understanding of health information sharing. Lastly, the study strives to surmount the limitations of traditional benchmark regression models that can only investigate a single path and are prone to measurement errors. Instead, a structural equation model (SEM) is employed to scrutinize the amalgamated paths through which influencing factors affect information-sharing behavior. This approach allows for a more comprehensive understanding of the complexity of health information sharing in online communities, offering actionable insights for boosting user engagement and information dissemination. By tackling these issues, this study aims to extend both theoretical and practical understandings of health information-sharing behavior in online health communities.

### 1.1. Research Model and Hypothesis Development

#### 1.1.1. Technology Acceptance Model

In 1989, Davis [25] proposed the Technology Acceptance Model (TAM) to study user acceptance of information systems. This paper adopts this theory to analyze the factors influencing online health community users’ health information-sharing behavior. The TAM has two main factors: perceived usefulness and perceived ease of use. In this study, perceived usefulness is considered as users’ perception of the benefits of health information sharing, while perceived ease of use represents the convenience of health information sharing for community users. At the same time, the TAM believes that user behavioral intention is determined by a combination of perceived usefulness and perceived ease of use through certain influencing factors. In this study, user behavioral intention refers to the strength of users’ subjective health information-sharing intention.

Moreover, related studies have shown that perceived trust plays an important role in promoting information sharing. Information-sharing behavior requires understanding and trust between information seekers and information sharers. Perceived trust can reduce the perceived risks and costs of personal information sharing and has become an important factor influencing users’ health information-sharing intention in the online environment. Therefore, this paper adds perceived trust as a factor influencing users’ health information-sharing behavior.

In summary, based on the TAM, this paper takes perceived ease of use, perceived usefulness, perceived trust, and health information-sharing intention as factors influencing health information-sharing behavior.

#### 1.1.2. Theory of Planned Behavior

The Theory of Planned Behavior (TPB) was first proposed by Ajzen [26] based on the Technology Acceptance Model (TAM) by adding variables such as attitude and perceived behavioral control and expanding the scope of the model to a more objective level. In this theory, attitude refers to the individual’s evaluation of the degree of preference for performing a specific behavior. In this study, health information-sharing attitude refers to whether the user likes to post and share health information, and whether the user is interested in joining discussions related to health information. Perceived behavioral control refers to the individual’s perception of the difficulty or ease of performing a specific behavior, reflecting the individual’s perception of factors that facilitate or hinder the behavior. This study focuses on the user’s perception of the factors that promote health information sharing as the measurement items of perceived behavioral control.

In summary, this study combines the TPB and TAM to determine six variables, including perceived ease of use, perceived usefulness, perceived behavioral control, health information-sharing attitude, perceived trust, and health information-sharing intention, as the influencing factors of health information-sharing behavior.

#### 1.1.3. “Knowledge-Attitude-Practice” Theory

The “ Knowledge-Attitude-Practice”(KAP) theory was first proposed by Gust [27]. This theory suggests that individuals follow a process of “Knowledge-Attitude-Practice” when making behavior decisions. Specifically, an individual’s cognition and knowledge form the basis for behavior decisions, which then shape their beliefs and attitudes towards the behavior, ultimately leading to the behavior itself. This study posits that the six factors of perceived ease of use, perceived usefulness, perceived behavior control, health information-sharing attitude, perceived trust, and health information-sharing intention will collectively influence health information-sharing behavior according to the “Knowledge-Attitude-Practice” process.

In the “KAP” theory, knowledge refers to the behavior information that an individual perceives and grasps, which forms the foundation for behavior occurrence. In this study, knowledge refers to the relevant information that users perceive about online health information sharing, including perceived ease of use, perceived usefulness, perceived behavior control, and perceived trust. Belief refers to the subjective cognition and intention states that are formed by internalizing knowledge and are motivational factors for the ultimate occurrence of a behavior. In this study, health information-sharing attitudes and sharing intentions align with the basic definition of belief. In the “KAP” theory, behavior is defined as health information-sharing behavior from the perspective of this study.

Based on the Technology Acceptance Model and the Theory of Planned Behavior, this study has identified the influencing factors of health information-sharing behavior and, guided by the “KAP” theory, has clarified the basic logic of their interactions. To obtain a more specific model of the influencing factors of health information-sharing behavior, further analysis of the relationships among the variables is needed.

#### 1.1.4. Analysis of Factors Affecting Health Information-Sharing Attitude

According to the basic principles of the Technology Acceptance Model, perceived ease of use and perceived usefulness have a significant impact on the formation of behavior attitudes. Starting from the definition of perceived ease of use, it can be clarified that perceived ease of use represents the costs that an individual needs to pay to adopt a certain behavior, which to some extent determines the individual’s attitude towards the behavior. This article believes that users’ perception of ease of use in online health communities is specifically manifested in the users’ belief that the operations in various sections of the communities are convenient, which can reduce the time and effort required for users to share information and promote users’ positive health information-sharing attitude.

At the same time, perceived usefulness can significantly influence behavior attitudes. This article believes that when users believe that sharing health information in the communities can bring positive feedback to themselves and benefit others who obtain the shared information, it can promote users’ positive health information-sharing attitude behaviors.

In addition, perceived trust has been shown to affect attitude formation. In the context of sharing health information, perceived trust primarily refers to users’ trust in the online health community’s ability to provide positive benefits for their health and their belief that personal information will not be leaked during the sharing process. As a result, users’ perceived trust can have a positive impact on their attitude toward sharing in online health communities.

Finally, the Theory of Planned Behavior suggests that perceived behavioral control refers to an individual’s perception of the ease or difficulty of performing a specific behavior. This study argues that users’ perceived behavioral control should include both internal and external factors. Internal factors refer to users’ subjective ability to collect, process, edit, and publish information, while external factors refer to the objective conditions provided by the communities to support health information sharing, such as guidance, information processing, information feedback, and privacy protection. Accordingly, the study suggests that if the external factors of perceived behavioral control are met, users’ attitudes toward online communities and sharing behavior will improve. Therefore, the following hypotheses are proposed:

**Hypothesis 1** **(H1).** *Perceived ease of use has a significant positive impact on health information-sharing attitude.*

**Hypothesis 2** **(H2).** *Perceived usefulness has a significant positive impact on health information-sharing attitude.*

**Hypothesis 3** **(H3).** *Perceived trust has a significant positive impact on health information-sharing attitude.*

**Hypothesis 4** **(H4).** *Perceived behavioral control has a significant positive impact on health information-sharing attitude.*

#### 1.1.5. Factors Affecting Perceived Usefulness

In the Technology Acceptance Model, perceived ease of use can indirectly influence behavioral intention through perceived usefulness. In the context of online health communities, it is believed that when users perceive the community platform as easy to use and the information-sharing process as simple, their perceived ease of use is satisfied, which in turn enhances the efficiency of their information-sharing and increases their perceived usefulness. Therefore, the following hypothesis is proposed:

**Hypothesis 5** **(H5).** *Perceived ease of use has a significant positive effect on perceived usefulness.*

#### 1.1.6. Analysis of Factors Influencing Health Information-Sharing Intention

According to the Theory of Planned Behavior and the Technology Acceptance Model, perceived usefulness and attitude toward behavior can both influence behavioral intention. For example, Suh [28] found that perceived usefulness positively affects usage intention in their study of mobile reading users. In this study, when online health community users perceive that sharing health information is beneficial for both them and other members, it promotes the health information-sharing intention.

Moreover, the Technology Acceptance Model suggests that attitudes toward behavior affect behavioral intention. In this study, we propose that the more positively users feel about sharing health information, the more likely they are to perceive it as beneficial for their own health and generate the intention to share.

Additionally, perceived trust has a positive effect on behavioral intention. For example, Chiu-Ping Hsu [29] showed that users trust organizations and are more intentional to share information. This study suggests that perceived trust refers to users’ trust in the community’s information processing capabilities and privacy protection methods, as well as trust in other community members’ commitments and declarations. Therefore, a good trust relationship has a positive effect on users’ health information-sharing intention.

Finally, from the perspective of the Theory of Planned Behavior, individual behavioral intention is also influenced by perceived behavioral control. In this study, we believe that perceived behavioral control in the decision-making process of whether to share health information includes both internal and external aspects. The external aspect, as discussed earlier, can positively promote users’ health information-sharing attitude. The internal aspect, however, can significantly affect users’ health information-sharing intention. Therefore, the following hypothesis is proposed:

**Hypothesis 6** **(H6).** *Perceived usefulness has a significant positive effect on the health information-sharing intention.*

**Hypothesis 7** **(H7).** *Health information-sharing attitude has a significant positive effect on the health information-sharing intention.*

**Hypothesis 8** **(H8).** *Perceived trust has a significant positive effect on the health information-sharing intention.*

**Hypothesis 9** **(H9).** *Perceived behavioral control has a significant positive effect on the health information-sharing intention.*

#### 1.1.7. Analysis of Factors Influencing Health Information-Sharing Behavior

Health information-sharing behavior is the focus of this study. Guided by the Theory of Planned Behavior, the Technology Acceptance Model, and the “Knowledge-Attitude-Practice” theory, factors such as perceived ease of use, perceived usefulness, perceived trust, health information-sharing attitude, perceived behavioral control, and health information-sharing intention will have direct or indirect impacts on health information-sharing behavior. This study posits that perceived behavioral control and health information-sharing intention are two variables that have a direct impact on health information-sharing behavior.

Firstly, in the Theory of Planned Behavior, perceived behavioral control refers to the perceived control and ease of implementing a specific behavior. When individuals perceive that their behavior is easy to control, they are more likely to practice that behavior given other conditions are met. Conversely, when individuals perceive that they have limited control over the time and resources needed to carry out the behavior, their practice of the behavior will be restricted.

Secondly, in the Theory of Planned Behavior, intention is a direct determinant of behavior. The stronger the individual’s intention, the greater the likelihood of implementing the behavior. Therefore, the following hypotheses are proposed:

**Hypothesis 10** **(H10).** *Perceived behavioral control has a significant positive impact on health information-sharing behavior.*

**Hypothesis 11** **(H11).** *Health information-sharing intention has a significant positive impact on health information-sharing behavior.*

**Figure 1:**
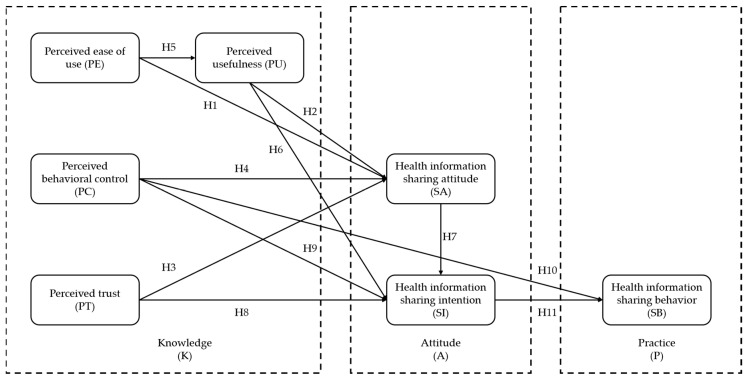
Health information-sharing behavior model of users in online health communities.

Based on the analysis of the above influencing factors and Hypotheses 1–11, a model of the influencing factors of health information-sharing behavior was constructed (see Figure 1).

## 2. Methods

### 2.1. Data Collection

In this study, a survey questionnaire was used to collect data on seven variables related to perceived ease of use, perceived usefulness, perceived behavioral control, health information-sharing attitude, perceived trust, health information-sharing intention, and health information-sharing behavior. The content of each measurement item was based on relevant measurement scales of the planned behavior theory and the technology acceptance model, as well as the research theme of this study. A total of 35 items were developed.

The Likert seven-point scale was used to evaluate the questionnaire, with each item containing seven options, ranging from 1 (strongly disagree) to 7 (strongly agree). After the initial development of the questionnaire, 15 individuals with experience using online health communities and five experts in the fields of health information and social media research were invited to provide feedback on the questionnaire’s content. Based on their feedback, some measurement item descriptions were modified, and an attention check question was included to ensure the validity of the questionnaire. Upon the collection of survey data, scores for each Likert item were recorded for every participant. Subsequently, factor analysis was employed to categorize these items into the aforementioned seven variables. Factor scores for each variable were computed for each participant utilizing factor loadings. This methodology facilitated a more precise reflection of the contribution of distinct items to the factor scores. Table 1 presents the specific content of each measurement item.

This survey was conducted online through various platforms, including the “Zhihu Health” and “Health Knowledge” topic modules on “Zhihu”, the “Disease Health Mutual Aid Group” and “Health Problem Discussion Group” groups on “Douban”, the “Health Home Bar” on “Baidu Tieba”, and the “Health” super topic, “Meet you” Q&A, and “Chunyu Doctor” on “Weibo”. We distributed the survey through links and QR codes and received a total of 621 responses. After screening the responses, we excluded the questionnaires that did not meet the requirements of the attention test question, resulting in a final sample of 573 valid responses. The effective response rate was 92.27%.

### 2.2. Data Analysis

#### 2.2.1. Exploratory Factor Analysis

To verify the validity of the questionnaire design, this study used SPSS26.0 and conducted an exploratory factor analysis to test the validity of the questionnaire design. The validity was analyzed comprehensively using indicators such as KMO (Kaiser–Meyer–Olkin) value, communality, and factor loading coefficient (see Table 2 for results). The KMO value was 0.933, which is significantly greater than 0.7. In Bartlett’s sphericity test, the approximate chi-square value was 13,540.698, and the *p*-value was less than 0.001. Therefore, the null hypothesis of Bartlett’s sphericity test was rejected, indicating that the validity structure of the questionnaire was good and could be subjected to factor analysis.

Furthermore, the communality of each questionnaire item was greater than 0.40, indicating that the indicators of each item significantly influenced the scale and should be retained. Finally, by rotating the component matrix of each item in the scale, the results showed that the loading values of each factor item were all greater than 0.7 and could be used as important items for analysis. The results of the rotated component matrix were consistent with the dimension of the designed scale. Therefore, the validity of the questionnaire was high, and the questionnaire was effective. In Table 2, Factor 1 corresponds to perceived usefulness, Factor 2 corresponds to health information-sharing behavior, Factor 3 corresponds to perceived ease of use, Factor 4 corresponds to perceived behavioral control, Factor 5 corresponds to perceived trust, Factor 6 corresponds to health information-sharing intention, and Factor 7 corresponds to health information-sharing attitude.

#### 2.2.2. Confirmatory Factor Analysis

To verify the reliability of the questionnaire data, this study conducted reliability and validity tests through confirmatory factor analysis using SPSS26.0. As shown in Table 3, Cronbach’s α coefficient values of each variable in the questionnaire are all greater than 0.7, and the CR values are all greater than 0.7, indicating that the questionnaire data have good reliability. Convergent validity and discriminant validity were tested, and Table 3 shows that the average extracted variance (AVE) of each variable is greater than 0.5, indicating that the variables have good convergent validity. Discriminant validity was measured by comparing the square root of AVE with the correlation coefficients between variables. As shown in Table 4, the square root of AVE for each variable is greater than the correlation coefficients between those variables and other variables, indicating that the variables have good discriminant validity.

#### 2.2.3. Model Verification

**Figure 2:**
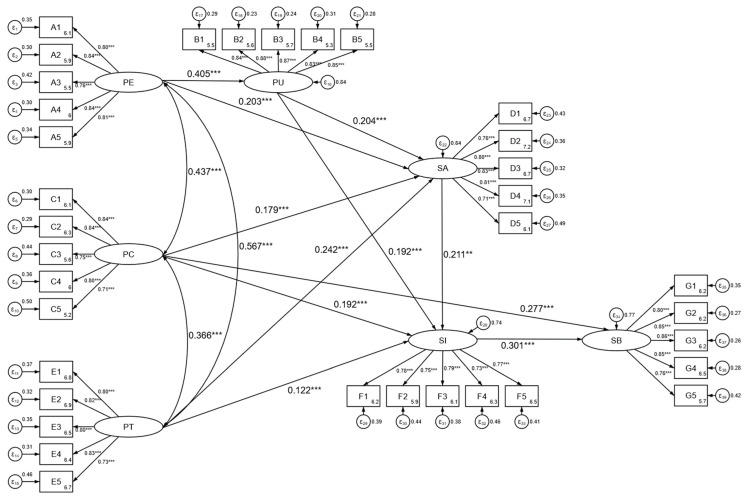
Path parameter estimation results of the structural equation model. *** represents a significance level of 1%, ** represents a significance level of 5%.

In this study, structural equation modeling analysis and verification were conducted using STATA15.1, with the specific results shown in Figure 2. The model fit results are shown in Table 5, indicating a good fit for the model. At the same time, the 11 hypotheses proposed were tested, and the results are shown in Table 6, with all hypotheses being supported.

Perceived ease of use (β = 0.203, *p* < 0.001), perceived usefulness (β = 0.204, *p* < 0.001), perceived trust (β = 0.242, *p* < 0.001), and perceived behavioral control (β = 0.179, *p* < 0.001) all had a significant positive impact on health information-sharing attitude, supporting hypotheses H1, H2, H3, and H4. Perceived ease of use (β = 0.405, *p* < 0.001) had a significant positive impact on perceived usefulness, supporting hypothesis H5. Perceived usefulness (β = 0.192, *p* < 0.001), health information-sharing attitude (β = 0.211, *p* < 0.001), perceived trust (β = 0.122, *p* = 0.019), and perceived behavioral control (β = 0.192, *p* < 0.001) all had a significant positive impact on health information-sharing intention, supporting hypotheses H6, H7, H8, and H9. Perceived behavioral control (β = 0.277, *p* < 0.001) and health information-sharing intention (β = 0.301, *p* < 0.001) both had a significant positive impact on health information-sharing behavior, supporting hypotheses H10 and H11.

### 2.3. Empirical Analysis Based on fsQCA

#### 2.3.1. Configuration Path Decomposition

QCA (Qualitative Comparative Analysis) was first introduced by Ragin [38] in the field of political science and social sciences. This method combines the strengths of both quantitative and qualitative research, emphasizing that complex sociological problems are formed by the combination and joint action of multiple factors, thus requiring the study of the configuration of multiple factors on the mechanism of problem outcomes. Among them, fuzzy set qualitative comparative analysis (fsQCA) sets the measurement range of variables to [0,1], and accurately assigns and objectively describes conditional variables. Given that user health information-sharing behavior itself has a certain degree of fuzziness, and the data studied in this paper were obtained through a questionnaire survey method, which has a certain degree of subjectivity, the fsQCA method is adopted to explore the configuration path of influencing factors on health information-sharing behavior of online health community users.

Based on Figure 1 and the hypotheses of this study, this article decomposes the configuration path of influencing factors on health information-sharing behavior into three sequential conditional configuration models: (1) Conditional Configuration Model A: with health information-sharing attitude as the outcome variable, perceived ease of use, perceived usefulness, perceived behavioral control, and perceived trust are selected as the antecedent variables; (2) Conditional Configuration Model B: with health information-sharing intention as the outcome variable, perceived ease of use, perceived usefulness, perceived behavioral control, perceived trust, and health information-sharing attitude are selected as the antecedent variables; (3) Conditional Configuration Model C: with health information-sharing behavior as the outcome variable, perceived ease of use, perceived usefulness, perceived behavioral control, perceived trust, health information-sharing attitude, and sharing intention are selected as the antecedent variables.

#### 2.3.2. Variable Assignment and Calibration

Before conducting a fuzzy-set qualitative comparative analysis, it is necessary to calibrate the antecedent variables and outcome variables and calibrate the sample data to the membership on the set (0,1). In this study, the factor analysis comprehensive score of the antecedent variables and outcome variables of each sample was used as the judgment standard for the QCA truth table. Following Ragin’s [39] standards, the 5% (fully out), 95% (fully in), and 50% (crossover point) were used as the corresponding percentile values using the Excel percentile function. Then, the calibration of the sample data was completed using the “calibrate” function of the fsQCA software, and the results are reported in Table 7.

#### 2.3.3. Necessity Analysis

Before conducting the configurational analysis, it is necessary to perform a necessity analysis on models A, B, and C to determine the necessity of individual independent variables as predictors of the dependent variable. The results are shown in Table 8. The results indicate that the consistency of the antecedent variables for health information-sharing attitude (SA), sharing intention (SI), and sharing behavior (SB) did not reach the standard of an absolutely necessary condition of 0.9. In other words, there is no antecedent variable that can serve as a necessary condition for health information-sharing attitude, sharing intention, and sharing behavior. Therefore, it is necessary to analyze sufficiency by combining multiple independent variables.

#### 2.3.4. Condition Configuration Analysis

This article used fsQCA 3.0 to solve the path of the conditional configuration models A, B, and C. Ragin [40] suggested that the consistency threshold for the configuration path detection should not be less than 0.75, and the natural break in sample consistency should be considered. Based on this principle, and to ensure the overall solution consistency remains at a high level, this article set the consistency threshold of models A and B to 0.8, and the consistency threshold of model C to 0.9 according to previous research. Since the total sample size of this study is 573, which belongs to a large sample size, it is necessary to set the case frequency to 2 [41] to retain more than 80% of the samples. Finally, to reduce the conflicts between the configurations, the PRI consistency threshold was set to 0.7 [42], to construct a truth table, and obtain the complex, parsimonious, and intermediate solutions for the configuration models A, B, and C (see Table 9 and Table 10).

Generally, it is believed that the independent variables that appear in both the simplified and intermediate solution of the same configuration are referred to as core conditions, while the independent variables that only appear in the intermediate solution and not in the simplified solution are referred to as peripheral conditions. Based on this, the results of the intermediate solution of the antecedent condition configuration for the health information-sharing attitude and intention are shown in Table 11, and the results of the intermediate solution of the antecedent condition configuration for the health information-sharing behavior are shown in Table 12.

There were two path configurations for each of the result variables, health information-sharing attitude and intention, with two necessary conditions for each. The consistency of the solutions for models A and B was 0.830939 and 0.862403, respectively, and the coverage rate was over 50% for both. A1 and A2 were “~perceived ease of use*perceived usefulness*~perceived behavioral control*~perceived trust” and “perceived ease of use*perceived usefulness*perceived behavioral control*perceived trust”, respectively, while B1 and B2 were “~perceived ease of use*perceived usefulness*~perceived behavioral control*~perceived trust*~health information-sharing attitude” and “perceived ease of us*perceived usefulness*perceived behavioral control*perceived trust*health information-sharing attitude”, respectively.

For the result variable of health information-sharing behavior, there were two path configurations, with three sub-paths for C1 and two for C2. C1a’s configuration was “perceived ease of use*perceived usefulness*perceived trust*health information-sharing attitude*health information-sharing intention,” C1b’s configuration was “~perceived ease of use~*perceived usefulness~*perceived behavioral control*perceived trust*~health information-sharing attitude*health information-sharing intention,” and C1c’s configuration was “perceived usefulness*perceived behavioral control*perceived trust*health information-sharing attitude*health information-sharing intention.”. C2a’s configuration was “perceived ease of use*perceived usefulness*perceived behavioral control*health information-sharing attitude*health information-sharing intention,” while C2c’s configuration was “perceived ease of use*perceived usefulness*perceived behavioral control*perceived trust*health information-sharing attitude”. The overall consistency of the solutions for model C reached 0.905978, exceeding the threshold of 0.8, and the overall coverage rate was 0.645424, indicating more than 64% explanatory power.

## 3. Result

### 3.1. Configuration Path Analysis with Health Information-Sharing Attitude and Health Information-Sharing Intention as Outcome Variables

Based on the characteristics and performance of the A1, A2, B1, and B2 configurations, these four paths can be classified into two types: perceived usefulness-driven and comprehensive perceived goodness.

(1) Perceived usefulness-driven: corresponds to paths A1 and B1. The core variables of these two paths are high perceived usefulness and low other antecedent variables. It can be seen that perceived usefulness plays a critical role in forming attitudes and health information-sharing intention in online health communities. Perceived usefulness drives users to form positive attitudes toward the communities and generate health information-sharing intention. When users perceive that sharing health information can improve their own benefits and produce positive externalities, even if the online health communities cannot bring users a convenient experience, high perceived behavioral control, and strong perceived trust, users will still generate health information-sharing attitudes and intentions. However, without perceived ease of use, perceived behavioral control, and perceived trust, this kind of health information-sharing attitude and intention may not necessarily be translated into sharing behavior.

(2) Comprehensive perceived goodness corresponds to paths A2 and B2. The core variables of these two paths are simultaneously high perceived ease of use, perceived usefulness, perceived behavioral control, and perceived trust. This path shows that under ideal conditions, perceived ease of use, perceived usefulness, perceived behavioral control, and perceived trust can jointly be transformed into health information-sharing attitudes and intention, which fully proves the correctness of hypotheses H1–H4 and H6, H8, and H9. At the same time, path B2 shows that high health information-sharing attitudes are also the core antecedent conditions for producing high health information-sharing intention, which further verifies hypothesis H7.

### 3.2. Configuration Path Analysis with Health Information-Sharing Behavior as Outcome Variable

According to the characteristics and performance of the subpaths C1a, C1b, C1c, C2a, and C2b, this paper categorizes path C1 as a high-trust-assisted path and path C2 as a comprehensive-advantage-empowering path. The high-trust-assisted path is derived from the perceived-use-driven path, while the comprehensive-advantage-empowering path is derived from the comprehensive-perception-good path.

(1) High-trust-assisted path corresponds to subpaths C1a, C1b, and C1c. These three subpaths indicate that regardless of whether there is a high perceived ease of use, high perceived usefulness, high perceived behavioral control, and high health information-sharing attitude, as long as users have a high level of perceived trust and health information-sharing intention towards online health communities, they can form health information-sharing behavior. According to the perceived-use-driven paths A1 and B1, the formation of health information-sharing intention requires a high level of perceived usefulness, but the configuration of paths A1 and B1 cannot ultimately convert health information-sharing intention into sharing behavior. At this time, users also need to have a high level of perceived trust in online health communities to enable users with high health information-sharing intention to engage in sharing behavior, thereby forming a high-trust-assisted path configuration.

(2) The comprehensive-advantage-empowering path corresponds to subpaths C2a and C2b. These two subpaths indicate that high perceived ease of use, perceived usefulness, perceived behavioral control, and health information-sharing attitude are necessary conditions for promoting health information-sharing behavior and that both perceived trust and health information-sharing intention must be present, either one of which can ultimately promote the formation of health information-sharing behavior. The C2 configuration shows that when the online health communities are well-operated, users are satisfied and will actively participate in health information sharing. The comprehensive-advantage-empowering path configuration proves the rationality of the influence factors set in this paper.

## 4. Discussion

### 4.1. Main Findings

Research employing structural equation modeling revealed that the intention to share health information and perceived behavioral control directly impacts health information-sharing behavior. This implies that individuals who are willing to share their health information and perceive that they have control over this behavior are more inclined to engage in health information sharing. Furthermore, perceived usefulness, perceived behavioral control, attitude toward sharing, and perceived trust significantly enhance the intention to share health information. This suggests that individuals who perceive health information sharing as beneficial, believe they can control this behavior, have a positive attitude towards it, and trust the recipient of their information, exhibit a stronger intention to share health information. Additionally, perceived ease of use, perceived usefulness, perceived behavioral control, and perceived trust positively influence the attitude toward health information sharing. This indicates that individuals who perceive health information sharing as easy and beneficial believe they can control this behavior, and trust the recipient of their information, and have a more positive attitude toward health information sharing. Lastly, perceived ease of use significantly boosts perceived usefulness. This implies that individuals who perceive health information sharing as easy are more likely to view this behavior as beneficial. Consequently, enhancing users’ perceived ease of use and perceived usefulness can strengthen their intention and attitude toward health information sharing. Similarly, improving users’ perceived behavioral control and perceived trust can effectively facilitate health information sharing.

Fuzzy set qualitative comparative analysis revealed two distinct modes of configuration paths, with health information-sharing attitude and health information-sharing intention as outcome variables. These are the perceived usefulness-driven mode and the comprehensive perceived goodness mode. In these modes, perceived usefulness emerges as a crucial antecedent variable. Ideally, the formation of a health information-sharing attitude and intention necessitates the synergistic effect of perceived ease of use, perceived behavioral control, and perceived trust. The antecedent configuration of health information-sharing attitude, when considered as the outcome variable, is influenced by two modes: the high-trust-assisted mode and the comprehensive-advantage-empowering mode. In the high-trust-assisted mode configuration path, perceived trust and health information-sharing intention are essential antecedent variables. Conversely, in the comprehensive-advantage-empowering mode configuration path, perceived usefulness, perceived behavioral control, and health information-sharing attitude are vital antecedent conditions for the formation of health information-sharing behavior. A notable distinction between the comprehensive-advantage-empowering path and the high-trust-assisted path is the potential absence of perceived trust and health information-sharing intention in the former. On one hand, the higher perceived ease of use, perceived usefulness, and perceived behavioral control in online health communities can compensate for the lack of perceived trust. Specifically, high perceived behavioral control can enhance an individual’s ability to perceive risk avoidance and control costs, thereby mitigating the risk perception caused by the lack of perceived trust. For instance, establishing a mechanism for retracting shared information in the community can reduce the risk posed by inadequate personal information protection. On the other hand, the antecedent condition configuration of the sub-path C2b aligns perfectly with the antecedent condition configuration of path B2, with health information-sharing intention as the outcome variable. Therefore, it can be inferred that the configuration of C2b itself can create the condition for health information-sharing intention, thus rendering health information-sharing intention potentially non-existent.

### 4.2. Theoretical Contributions and Practical Implications

This study contributes to theoretical advancements by validating and extending existing models such as the Technology Acceptance Model and the Theory of Planned Behavior within the context of online health communities. Distinctively, the research synergizes these prevalent theories with the “Knowledge-Attitude-Practice” paradigm, thus refining our understanding of the multifaceted dynamics influencing health information sharing. Furthermore, by identifying and dissecting unique configuration paths, this study illuminates the variable dynamics involved, paving the way for a more nuanced conceptualization of online health information-sharing behavior, thereby enriching the theoretical framework in this area.

On a practical level, the study provides actionable insights conducive to shaping user engagement strategies in online health communities. The significance of perceived ease of use, perceived usefulness, and perceived behavioral control in molding health information-sharing behavior suggest that enhancements to these factors should be the focus of interventions. For example, user interface improvements could enhance ease of use, while training could bolster perceived behavioral control. The pivotal role of trust, as identified in this research, underlines the importance of rigorous data protection measures to establish user trust and promote health information sharing. Furthermore, the unique configuration paths discovered offer a potential diagnostic tool for community administrators to identify key areas for encouraging health information sharing.

## 5. Conclusions

This research conducts a comprehensive examination of online health community users, utilizing the Technology Acceptance Model, Theory of Planned Behavior, and “Knowledge-Attitude-Practice” theory as guiding frameworks. The study validates the impact of perceived ease of use, perceived usefulness, perceived behavioral control, health information-sharing attitude, perceived trust, and health information-sharing intention on health information-sharing behavior, employing Structural Equation Modeling. Furthermore, the research utilizes the three-stage fuzzy set Qualitative Comparative Analysis method to discern the antecedent configurations of user health information-sharing behavior. The insights from this research enrich the existing literature by offering a detailed understanding of the determinants influencing health information-sharing behavior in online communities. The study emphasizes the significance of perceived ease of use, perceived usefulness, and perceived behavioral control in molding users’ attitudes and intentions toward health information sharing. Additionally, it underscores the pivotal role of trust in fostering health information-sharing behavior.

As a future direction, subsequent research can build upon this study by investigating the impact factors of health information-sharing behavior grounded in other theoretical models, such as the Stimulus-Organism-Response (SOR) model and the DeLone and McLean Information System Success Model (D&M). Such explorations can aid in unveiling the specific mechanisms that form perceived ease of use, perceived usefulness, and other knowledge-related factors. They can also augment our comprehension of the interaction between these influencing factors, thereby rendering a more holistic view of health information-sharing behavior in online communities. This, in turn, can guide the formulation of strategies and interventions aimed at encouraging health information sharing, ultimately leading to enhanced health outcomes and the progression of health-related knowledge.

## Figures and Tables

**Table 1:** Measurement items for the survey questionnaire.

Serial Number	Variable	Measurement Item Content	Source
1	Perceived ease of use (PE)	A1. The way of sharing health information in online health communities is easy to learn and does not take too much time. A2. The interface design of online health communities is user-friendly and easy to understand. A3. The health information shared in the communities is highly relevant to the topic and easy to share. A4. Sharing health information in online health communities is quick and easy for me. A5. Editing and sharing health information in online health communities is easy, and I can express the information I want to share clearly through text, images, videos, and other means.	Hansen J M [30], Koufaris M [31]
2	Perceived usefulness (PU)	B1. Sharing health information in the communities can help me solve some health problems. B2. Sharing health information in the communities allows me to obtain comments and feedback from other users and experts. B3. Health information in the communities broadens my relevant knowledge. B4. Most of the health information pushed by the communities is what I need. B5. High-quality information that I share in the communities will be promptly pushed by the communities.	Rese A [32], Venkatesh and Davis [33]
3	Perceived behavioral control (PC)	C1. Whether or not I share health information in the communities depends entirely on me. C2. I am confident that I can share health information in the communities. C3. The communities provide me with all the necessary conditions for sharing health information online. C4. I can withdraw the health information I shared in the communities at any time. C5. I can decide who to share my health information with.	Yoon C [34]
4	Health information-sharing attitude (SA)	D1. I am willing to share health information in the online health communities. D2. Sharing health information in the communities gives me pleasure. D3. I think sharing health information in the online health communities can help others. D4. I think sharing health information in the online health communities can benefit me. D5. I will continue to choose the online health communities to deal with my health problems.	Wang W T [35], Venkatesh and Davis [33]
5	Perceived trust (PT)	E1. I believe the communities are trustworthy and will not disclose my personal information at will. E2. I believe the communities have the ability to ensure the authenticity of health information in the communities. E3. I believe that the health information shared among community members is trustworthy. E4. I trust that the online health communities have the ability to provide me with useful health information. E5. I can choose not to provide personal information that I do not want to provide (such as the communities’ anonymous system).	Oum S [36], McKnight [37]
6	Health information-sharing intention (SI)	F1. I am willing to share health information through the online health communities. F2. When I encounter problems, I am willing to share my health information to get advice on solving them. F3. I am willing to forward and disseminate health information shared by others. F4. I agree with the way and process of sharing health information in the online health communities. F5. I am willing to recommend others to use the online health communities to share health information.	Venkatesh Davis [33]
7	Health information-sharing behavior (SB)	G1. I often browse health information in the communities and continue to pay attention to some shared information. G2. I often share health information in the communities, such as health problems, treatment methods, and experiences. G3. When discussing related health issues with community members, I will continue to participate in the discussion. G4. I often comment and forward health information in the communities. G5. When encountering problems, I often seek help through the communities.	Yan Z J [12]

**Table 2:** Results of exploratory factor analysis.

Name	Factor Loading Coefficient							Communality
	Factor 1	Factor 2	Factor 3	Factor 4	Factor 5	Factor 6	Factor 7	
A1			0.754					0.706
A2			0.819					0.772
A3			0.774					0.687
A4			0.767					0.746
A5			0.789					0.733
B1	0.830							0.772
B2	0.852							0.813
B3	0.845							0.802
B4	0.805							0.757
B5	0.841							0.789
C1				0.811				0.741
C2				0.859				0.793
C3				0.741				0.650
C4				0.805				0.715
C5				0.744				0.613
D1							0.702	0.647
D2							0.754	0.699
D3							0.777	0.743
D4							0.780	0.736
D5							0.786	0.663
E1					0.752			0.707
E2					0.799			0.744
E3					0.766			0.716
E4					0.789			0.747
E5					0.740			0.648
F1						0.787		0.694
F2						0.771		0.667
F3						0.776		0.702
F4						0.776		0.658
F5						0.783		0.675
G1		0.770						0.719
G2		0.816						0.774
G3		0.801						0.787
G4		0.805						0.772
G5		0.802						0.705
Eigenvalue	3.937	3.728	3.622	3.555	3.553	3.462	3.435	-
Variance explained ratio	11.248%	10.651%	10.349%	10.158%	10.150%	9.890%	9.815%	-
Cumulative variance explained ratio	11.248%	21.899%	32.248%	42.406%	52.556%	62.447%	72.262%	-
KMO measure	0.933							-
Bartlett’s sphericity test	13,540.698							-
df	595							-
*p*-value	***							-

*** *p* < 0.001.

**Table 3:** Results of confirmatory factor analysis.

Variable	Cronbach’s α	AVE	CR
Perceived ease of use	0.905	0.659	0.906
Perceived usefulness	0.931	0.730	0.931
Perceived behavioral control	0.887	0.622	0.891
Health information-sharing attitude	0.886	0.615	0.889
Perceived trust	0.897	0.637	0.897
Health information-sharing intention	0.878	0.592	0.879
Health information-sharing behavior	0.914	0.685	0.916

**Table 4:** Discriminant validity: Pearson correlations and the square root of AVE.

Variable	PE	PU	PC	SA	PT	SI	SB
Perceived ease of use (PE)	0.812						
Perceived usefulness (PU)	0.352	0.855					
Perceived behavioral control (PC)	0.382	0.276	0.789				
Health information-sharing attitude (SA)	0.432	0.392	0.368	0.784			
Perceived trust (PT)	0.498	0.408	0.316	0.447	0.798		
Health information-sharing intention (SI)	0.366	0.339	0.333	0.364	0.317	0.769	
Health information-sharing behavior (SB)	0.414	0.385	0.341	0.437	0.454	0.339	0.828

**Table 5:** Fit indices of the model.

Fit Indices	χ2	df	χ2/df	GFI	RMSEA	CFI	NFI	NNFI
Optimal standard values	-	-	<3	>0.9	<0.10	>0.9	>0.9	>0.9
Statistical values	1130.672	545	2.075	0.900	0.043	0.956	0.918	0.952
Fit status	-	-	Ideal	Ideal	Ideal	Ideal	Ideal	Ideal

**Table 6:** Results of hypothesis testing.

Hypothesis	X→Y	Non-Standardized Regression Coefficient (N-β)	Standardized Regression Coefficient (β)	Standard Error (SE)	z (CR Value)	*p*
Hypothesis 1	Perceived ease of use→Health information-sharing attitude	0.172	0.203	0.047	3.624	***
Hypothesis 2	Perceived usefulness→Health information-sharing attitude	0.152	0.204	0.032	4.697	***
Hypothesis 3	Perceived trustworthiness→Health information-sharing attitude	0.234	0.242	0.049	4.739	***
Hypothesis 4	Perceived behavioral control→Health information-sharing attitude	0.146	0.179	0.037	3.944	***
Hypothesis 5	Perceived ease of use→Perceived usefulness	0.458	0.405	0.051	9.000	***
Hypothesis 6	Perceived usefulness→Health information-sharing intention	0.162	0.192	0.038	4.228	***
Hypothesis 7	Health information-sharing attitude→Health information-sharing intention	0.237	0.211	0.061	3.868	***
Hypothesis 8	Perceived trustworthiness→Health information-sharing intention	0.132	0.122	0.054	2.433	0.019
Hypothesis 9	Perceived behavioral control→Health information-sharing intention	0.175	0.192	0.044	3.986	***
Hypothesis 10	Perceived behavioral control→Health information-sharing behavior	0.254	0.277	0.042	5.984	***
Hypothesis 11	Health information-sharing intention→Health information-sharing behavior	0.302	0.301	0.048	6.35	***

*** *p* < 0.001.

**Table 7:** Descriptive Statistics and Calibration Thresholds for Explanatory and Outcome Variables.

Variable	Descriptive Statistics of Variables				Calibration Threshold		
	Mean	Standard Deviation	Minimum Value	Maximum Value	Fully Membership	Crossover Point	Fully Non-Membership
PE	0	1	−5.557	1.873	1.803	0.083	−1.920
PU	0	1	−5.569	2.049	1.601	0.243	−1.767
PC	0	1	−5.948	1.937	1.520	0.172	−1.584
SA	0	1	−3.861	1.786	1.639	0.144	−1.587
PT	0	1	−5.549	1.643	1.658	0.241	−1.978
SI	0	1	−4.902	1.600	1.517	0.146	−2.034
SB	0	1	−5.382	1.803	1.352	0.326	−1.974

**Table 8:** Results of necessary condition analysis.

Dependent Variable	Health Information-Sharing Attitude (SA)		Health Information-Sharing Intention (SI)		Health Information-Sharing Behavior (SB)	
	Consistency	Coverage	Consistency	Coverage	Consistency	Coverage
PE	0.774890	0.746666	0.752731	0.760490	0.814127	0.784273
~PE	0.592860	0.573944	0.616449	0.778862	0.596409	0.577233
PU	0.745474	0.759891	0.728743	0.778862	0.798801	0.814042
~PU	0.627480	0.575813	0.640231	0.616007	0.626086	0.574387
PC	0.746304	0.741163	0.735222	0.765568	0.764315	0.758856
~PC	0.609699	0.573123	0.616793	0.607909	0.614482	0.577471
PT	0.762169	0.769400	0.724123	0.766444	0.799668	0.807048
~PT	0.611506	0.566128	0.651088	0.632005	0.609891	0.564489
SA	-	-	0.729708	0.765097	0.769160	0.768963
~SA	-	-	0.607245	0.594621	0.599625	0.559858
SI	-	-	-	-	0.780691	0.744391
~SI	-	-	-	-	0.604867	0.591545

**Table 9:** Configuration of explanatory variables for health information-sharing attitude and intention.

Dependent Variable	Solution	Combination of Conditions	Raw Coverage	Unique Coverage	Consistency	Coverage of Solution	Consistency of Solution
SA	Complex solution	~PE * PU * ~PC * ~PT	0.355643	0.0801566	0.811764	0.601966	0.830939
		PE * PU * PC * PT	0.521809	0.246323	0.885611		
	Parsimonious solution	~PE * PU * ~PC * ~PT	0.355643	0.0801566	0.811764	0.601966	0.830939
		PE * PU * PC * PT	0.521809	0.246323	0.885611		
	Intermediate solution	~PE * PU * ~PC * ~PT	0.355643	0.0801566	0.811764	0.601966	0.830939
		PE * PU * PC * PT	0.521809	0.246323	0.885611		
SI	Complex solution	~PE * PU * ~PC * ~PT * ~SA	0.334712	0.103057	0.872194	0.548926	0.862403
		PE * PU * PC * PT * SA	0.445869	0.214214	0.895907		
	Parsimonious solution	~PE * PU * ~PC * ~PT * ~SA	0.334712	0.103057	0.872194	0.548926	0.862403
		PE * PU * PC * PT * SA	0.445869	0.214214	0.895907		
	Intermediate solution	~PE * PU * ~PC * ~PT * ~SA	0.334712	0.103057	0.872194	0.548926	0.862403
		PE * PU * PC * PT * SA	0.445869	0.214214	0.895907		

**Table 10:** Configuration of explanatory variables for health Information-sharing behavior.

Dependent Variable	Solution	Combination of Conditions	Raw Coverage	Unique Coverage	Consistency	Coverage of Solution	Consistency of Solution
SB	Complex solution	PE * PU * SA * PT * SI	0.491795	0.0287377	0.944923	0.645424	0.905978
		PE * PU * PC * SA * PT	0.493819	0.0493057	0.946118		
		PE * PU * PC * SA * SI	0.467395	0.0228819	0.939475		
		PU * PC * PT * SA * SI	0.472456	0.019556	0.943478		
		~PE * ~PU * ~PC * ~SA * PT * SI	0.311705	0.0534987	0.905777		
	Parsimonious solution	PE * PU * SA * PT * SI	0.491795	0.056716	0.902363	0.648642	0.903389
		PE * PU * PC * SA * PT	0.493819	0.0493056	0.946118		
		PE * PU * PC * SA * SI	0.467395	0.022882	0.939475		
		PU * PC * PT * SA * S	0.472456	0.0175318	0.943478		
		~PE * ~PU * ~PC * PT * SI	0.338093	0.056716	0.902363		
	Intermediate solution	PE * PU * SA * PT * SI	0.491795	0.0287377	0.944923	0.645424	0.905978
		PE * PU * PC * SA * PT	0.493819	0.0493057	0.946118		
		PE * PU * PC * SA * SI	0.467395	0.0228819	0.939475		
		PU * PC * PT * SA * SI	0.472456	0.019556	0.943478		
		~PE * ~PU * ~PC * ~SA * PT * SI	0.311705	0.0534987	0.905777		

**Table 11:** Configuration of explanatory conditions for health information-sharing attitude and intention.

Dependent Variable	Health Information-Sharing Attitude		Health Information-Sharing Intention	
configuration	A1	A2	B1	B2
Perceived ease of use	⊗	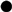	⊗	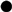
Perceived usefulness	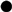	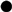	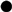	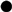
Perceived behavioral control	⊗	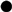	⊗	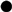
Perceived trust	⊗	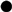	⊗	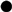
Health information-sharing attitude	-	-	⊗	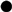
Consistency	0.811764	0.885611	0.872194	0.895907
Raw coverage	0.355643	0.521809	0.334712	0.445869
Unique coverage	0.0801566	0.246323	0.103057	0.214214
overall consistency	0.830939		0.862403	
overall coverage	0.601966		0.548926	

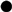
 represents the presence of core condition; ⊗ represents the absence of core condition; “Blank” represents that the existence or absence of the condition is uncertain.

**Table 12:** Configuration of explanatory conditions for health information-sharing behavior.

Configuration	C1a	C1b	C1c	C2a	C2b
Perceived ease of use	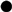	⭙		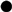	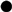
Perceived usefulness	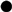	⭙	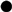	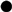	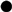
Perceived behavioral control		⭙	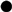	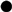	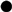
Perceived trust	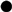	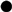	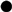		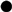
Health information-sharing attitude	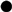	⨂	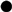	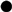	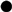
Health information-sharing intention	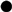	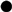	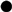	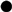	
Consistency	0.944923	0.905777	0.943478	0.939475	0.946118
Coverage	0.491795	0.311705	0.472456	0.467395	0.493819
Net coverage	0.0287377	0.0534987	0.019556	0.0228819	0.0493057
overall consistency	0.905978				
overall coverage	0.645424				

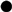
 represents the presence of core condition; ⭙ represents the absence of core condition; ⨂ represents the absence of marginal condition; “Blank” represents that the existence or absence of the condition is uncertain.

## Data Availability

The dataset presented in this research is available with a legitimate request from the corresponding author.
